# Challenges and needs for China to eliminate rabies

**DOI:** 10.1186/2049-9957-2-23

**Published:** 2013-10-02

**Authors:** Wenwu Yin, Jie Dong, Changchun Tu, John Edwards, Fusheng Guo, Hang Zhou, Hongjie Yu, Sirenda Vong

**Affiliations:** 1 Division of Infectious Diseases, Chinese Center for Disease Control and Prevention, Beijing, China; 2 Key Laboratory of Surveillance and Early-warning on Infectious Disease, Chinese Center for Disease Control and Prevention, Beijing, China; 3 World Health Organization, Country Office, Beijing, China; 4 OIE Reference Laboratory for Rabies, Institute of Military Veterinary, Academy of Military Medical Sciences, Changchun, China; 5 Food and Agriculture Organization, Beijing, China; 6 National Institute for viral disease control and prevention, Chinese Center for Disease Control and Prevention, Beijing, China

**Keywords:** Rabies, Elimination, China, Needs, Challenges, Review

## Abstract

In China, rabies is a significant public health concern where dogs remain the main reservoir of disease transmission to humans; rabies-related mortality ranks second in the world.

We compiled all published articles and official documents on rabies in mainland China to examine challenges and needs to eliminate rabies in the country. The Chinese authorities have identified rabies as a priority, recognized rabies control in dogs as key to control rabies in humans and required intersectoral collaborations. Efforts have been made to respond effectively to the latest re-emergence of rabies, which peaked in 2007 with >3,300 cases. Despite these outcomes and the increasing volume of publications and regulations in the recent years, our review points to some major information gaps to improve rabies control activities and envisage elimination program. An emphasis on laboratory or pathogen-associated and basic epidemiology research in the literature has contrasted with the absence of information to monitor various systems in humans and animals (e.g. quality of surveillance, response and post-exposure prophylaxis). Information is also lacking to appropriately inform policymakers (e.g. economic disease burden, impact of policies) and assist program managers (e.g. comprehensive and strategic guidance for cost-effective prevention and control activities, public education and dog population management).

In conclusion, strategic planning is needed to provide a sense of direction, demonstrate feasibility of elimination in China, and develop a research agenda, addressing country’s operational needs and constraints. The planning should be a multisectoral effort.

## Multilingual abstracts

Please see Additional file 1 for translations of the abstract into the six official working languages of the United Nations.

## Introduction

Despite the existence of an effective vaccine, rabies remains a public health problem worldwide, especially in developing countries. Around 3.9 billion people are at-risk, and approximately 55,000 people die of rabies each year worldwide and >150 countries are affected. China is a high-risk environment for rabies, with the number of related deaths being second only to India and where dogs continue to serve as the main reservoir of disease transmission to humans [1]. For the past decade, China has faced a reemergence of the disease, rabies being currently reported among the top three causes of human death due to infectious diseases [2].

There has been evidence that canine rabies elimination is feasible in many countries [3-7]. This has happened through implementation of strict dog population control measures and synchronized mass dog vaccination, in Central and South America [8]. In Asia, rabies has been controlled or eliminated for decades in Malaysia, Japan and many island countries or regions [5]. There is a growing global momentum for elimination of canine rabies through the formation of the *Global Alliance for Rabies Control* and the *Partners for Rabies Prevention Group*[9]. In South Asia, many recent efforts have been made for canine rabies elimination through coalitions and partnerships between regional institutions, as recommended by the FAO and WHO. The Association of Southeast Asian Nations (ASEAN) and the South Asian Association for Regional Cooperation (SAARC) adopted the resolution to prevent and control rabies, with the goal of rabies elimination by year 2020 [9]. It is widely recognized that rabies elimination requires an integrated approach by animal and human related services. There is growing support for the use of progressive control pathway strategy to achieve elimination of rabies (de Balogh K, unpublished observation). This strategy is particularly well suited to countries that are large and diverse. We reviewed the literature to assess the current situation in China with a special of focus on identifying future needs to control human rabies through eliminating canine rabies. This report describes the results of this work.

## Review

### Methods

#### Rabies Advisory and Technical Board (RATB)

Under the United National Theme Group on Health [10] Sub-working Group on Diseases at the Human-Animal Interface in China, the World Health Organization (WHO) and Food and Agriculture Organization (FAO) have sponsored and served as Secretariats of a Board which consists of independent national rabies specialists in public health, veterinary science, clinical medicine, and laboratory sciences. These experts are in-country advocates for best practices and research in rabies control and elimination in China. The Board meets twice a year to assess various aspects of rabies prevention and control in the country [11].

#### Guiding principles for rabies elimination

Rabies virus belongs to the lyssavirus genus, which also includes other lyssavirus species that cause rabies-like diseases and can be carried by domestic animals, wild carnivores and many bats species. However, the rabies virus is the only pathogen responsible for the large majority of rabies cases in humans. Of utmost importance, this virus is typically perpetuated solely by domestic dogs, which makes elimination of rabies virus realistic [4,6]. Therefore the paradigm of rabies control in humans is driven by interrupting rabies virus circulation in the dog population through mass vaccination (i.e. aim to achieve 70% vaccination coverage threshold) [12]. Elimination of rabies is feasible and cost effective [4,7,13]. The progressive roadmap to reaching elimination status lies on three pillars. The first one is the control of rabies in dogs; the second is to provide full access to adequate post-exposure prophylaxis (PEP) in humans, and lastly is to ensure effective disease and animal reservoir surveillance throughout the course of the campaign. Rabies elimination occurs solely through a multisectoral approach and above all, effective implementation requires sustained political commitment from all levels and sectors of the government; integration of the health, livestock and local government system; and through the partnership and involvement of community members and those engaged with education and the environment.

#### Literature review

We searched (1) the Pubmed database (1963–2012) using the following search terms “rabies” [MeSH Terms] OR “rabies” [All Fields]) OR “animal bites” [Titles/Abstracts] AND China [Affiliation] NOT Taiwan [Affiliation]) NOT Hong Kong [Affiliation]) NOT Macau [Affiliation] and (2)wanfang.med.online, a Chinese Database (3)baidu.com, a Chinese search-engine, and (4) official government websites using the same keywords. We manually screened titles and abstracts to exclude all papers related to non-death associated animal bites, rabies virus basic microbiology and rabies virus as gene therapy vectors. The full texts of the eligible papers were accessed. We also contacted officials from the Ministries of Health and Agriculture and other agencies involved in rabies-related activities and requested national reports/guidelines, proceedings and unpublished observations (oral presentations).

In this report, we analyzed the Chinese rabies situation and presented the results of the review using the following categories: (1) epidemiological situation; (2) level of awareness; (3) control in humans; (4) prevention and control in animals and; (5) surveillance in humans and dogs.

## Results

The literature search identified 201 reports of interest including 189 articles from Pubmed and 12 official documents (guidelines/protocols, legislative documents, and official notices/reports).

**Figure 1:**
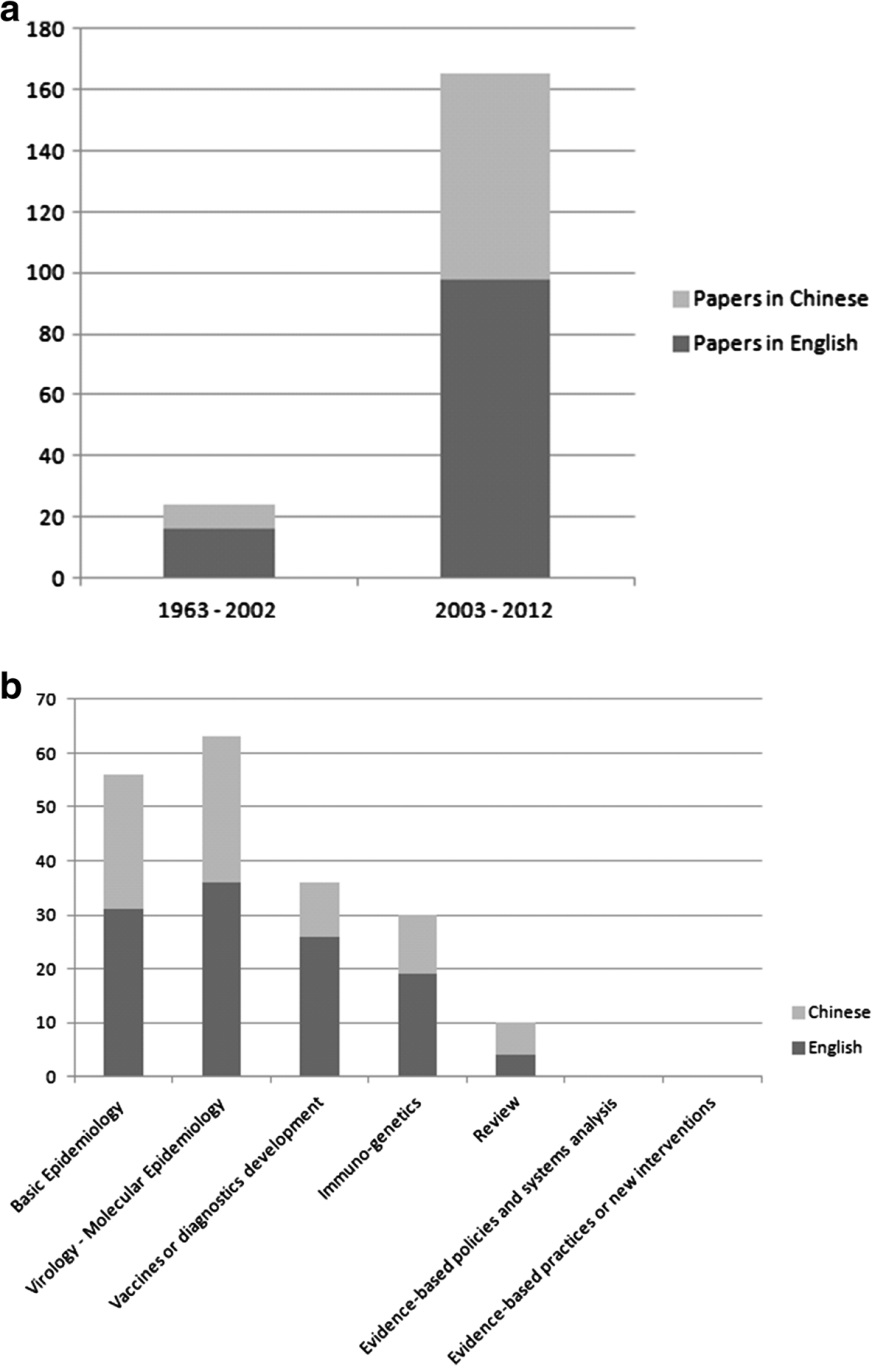
**Rabies related publications in China, 1963-2012. (a)** categorized by period; **(b)** categorized by type of outputs.

Scientific publications on rabies have increased by tenfold in China since 2003, particularly in English language (n = 114, 60.3%) journals. This indicates renewed attention given to a commonly neglected disease (Figure 1a), probably due to the latest disease re-emergence and increased international interest. The different categories of rabies-related publications are presented in Table 1. The key results included the contrast between an emphasis on laboratory or pathogen-associated and basic epidemiology research and the lack of research (1) for effective policies, (2) to analyze system-wide issues (surveillance, response, PEP clinics quality and coverage) or (3) improve existing control interventions in humans and animals (Figure 1b).

**Table 1:** Distribution of rabies-related scientific articles from Mainland China by category group, 1963 – 2012

	**Scientific articles**			
	**In English, n = 114**	**In Chinese, n = 75**	**Total, N = 189**	**%**
**Journal type**				
General	101	16	117	61.9%
Human	11	59	70	37.0%
Animal	2	0	2	1.1%
**Sector**				
Human	56	61	117	61.9%
Animal	36	4	40	21.2%
General	22	10	32	16.9%
**Setting**				
Lab-based	86	49	135	71.4%
Clinical/facility-based	15	8	23	12.2%
Urban community based	1	3	4	2.1%
Urban-Rural (mixed)	11	14	25	13.2%
Occupational	0	0	0	0.0%
Rural community based	1	1	2	1.1%
**Species**				
Pathogen	59	37	96	50.8%
Human	30	29	59	31.2%
Dog	11	3	14	7.4%
Multiple animals	14	6	20	10.6%
**Immuno-genetics**	**19**	**11**	**30**	
**Basic epidemiology**	36	27	63	**100.0%**
Case studies or outbreak reports in humans and animals	17	4	21	33.3%
Seroprevalence in animals	3	4	7	11.1%
Surveillance data and risk factors	6	14	20	31.7%
*PEP patients’ analysis	9	1	10	15.9%
Costs of treatment	0	1	1	1.6%
Level of awareness in public and health care workers	1	3	4	6.3%
**Vaccines and diagnostics development**	**26**	**10**	**36**	
**Virology - Molecular epidemiology**	30	20	50	**100.0%**
Virology	11	15	26	52.0%
Molecular epidemiology	14	3	17	34.0%
Modeling - dynamics	5	2	7	14.0%
**Review**	**4**	**6**	**10**	
**Evidence based policies and systems analysis**	**0**	**0**	**0**	
**Evidence-based practices or new interventions**				
Dogs management	**0**	**0**	**0**	
Caracteristics of dogs: pop. estimates/of owned	**0**	**0**	**0**	
Development of Monitoring and Evaluation tools	**0**	**0**	**0**	
**Evidence-based advocacy**				
Disease burden - incidence	**0**	**0**	**0**	
Cost of illness	**0**	**0**	**0**	

*PEP: Post-exposure prophylaxis.

### Epidemiological situation

**Figure 2:**
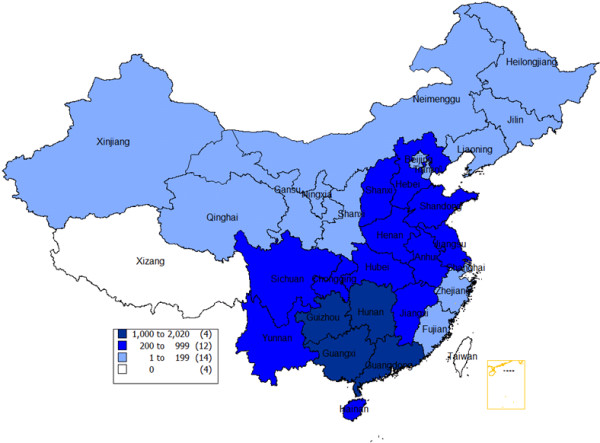
Number of human rabies cases reported in China during 2007–2012.

The latest resurgence of human rabies cases occurred in 1997 – 2001, notably in southern and eastern provinces, reaching a peak of 3,300 cases in 2007 (Figure 2). In 2011, 86% of the human cases lived from farming families in rural area including farmers (69.5%) and children (16.5%). Patients died of rabies mostly from dog (95%) and cat (4%) bites. Of these, 77% did not have wound cleaning in clinics and 91% did not have timely or appropriate PEP. Most (51%) human cases were reported from five southern provinces (i.e. Guizhou, Guangxi, Hunan, Guangdong and Yunnan) [2].

In China, all reports of *lyssavirus* isolates but one (i.e. non-RABV virus in bats in Jilin Province) [14] have been RABV, of which all were from the classic genotype I, which pertains to viruses found in dogs on all continents. Although wildlife rabies investigation is quite limited in China, it is thought that wildlife rabies (e.g. in ferret badgers) stem from spillover from dogs [15-17]; however, some strains isolated from ferret badgers were only identified in limited areas of Zhejiang and Jiangsu provinces showed only 89% of phylogenetic similarity with local dog viruses and was closer to a dog variant of Southern China [18]. This could be an independent wildlife cycle that could preclude country-wide elimination [18,19]. Investigations in the wildlife, which also include bats need to continue.

In China, authors believed that rabies epidemic may have a 10 year-cycle, and that reductions can be attributed to dog depopulation rather than dog mass vaccination [15,20]. The re-emergence is believed to be caused by low vaccination coverage in dogs and is coincidental with many socio-economic changes in the country [21]. First, the fiscal decentralization and privatization of the heath care system has led to lower access to health care services for many poor and rural families [22]; second, the uncontrolled dog population growth, particularly in rural areas; third, the development of the transportation network which increases movement of people with their dogs; and fourth, the replacement of concentrated to more expensive purified cell-culture vaccines [23]. A steady decline in incidence has occurred since 2008; there were 1,425 cases in 2012 (57% in 2007), particularly in endemic provinces (42%, 51% and 40% in 2007 in Guangxi, Guizhou and Guangdong provinces respectively). Documents that analysed the determinants of this decrease were not found.

### Level of awareness

Awareness among policymakers has risen since the rapid and steady increase of human rabies case reports in the past decade (Table 2). The State Council issued official notices in 2009 and 2012 underlining rabies control as a priority and providing program control objectives between 2015 and 2020 [24,25]. Specific rabies-related regulations on roles, responsibilities, and multisectorial cooperation were passed in recent years between the following four ministries [26]. There are (1) the Ministry of Public Security is responsible for the registration of dogs and management, killing of illegal, stray and wild dogs, (2) the Ministry of Agriculture (MoA) for rabid dog control and prevention (vaccination, quarantine), surveillance and response, (3) the State’s Food and Drugs Administration for vaccine prequalification and (4) the National Health and Family Planning Commission (NHFPC) for surveillance, PEP and vaccine/immunoglobulin supply in humans. Following these regulations in 2005 the NHFPC and MoA established a cooperation mechanism in China [27].

**Table 2:** Laws, regulations and technical documents regarding rabies control issued by China since 1990

**Type of official documents**	**Source**	**Year**	**Remarks**
**Legislations**			
Law on the Prevention and Control of Infectious Diseases	Standing Committee of the National People’s Congress	2004	Included rabies as a priority
The Enforcement Measures for Law on the Prevention and Control of Infectious Diseases	Approved by State Council, issued by NHFPC	1991	Roles & responsibilities of different departments and levels
People’s Republic of China on Animal Epidemic Prevention	The Standing Committee of the National People’s Congress	2008	Similar with the Law on the prevention and control of infectious diseases with focus on principles for animal epidemic prevention.
**Regulations and technical documents**			
Rabies Post-Exposure Treatment Standard	NHFPC*	2009	Requirements for post-exposure prophylaxis
National Rabies Surveillance Program	NHFPC	2005	Guidelines for national surveillance in humans
Present Situation of Rabies Prevention and Control in China	NHFPC, MoA, MoPS and SFDA	2009	Updates of rabies situation in China
Diagnostic Criteria for Rabies	NHFPC	2008	Guidelines for laboratory diagnosis
Cooperation Mechanism of Zoonosis Diseases Prevention and Control among NHFPC and MOA	NHFPC and MoA	2005	Work coordination group across ministries. Director-Generals for lead roles. Information sharing, meetings, joint outbreak investigations
Notice on Strengthening Rabies Prevention and Control	NHFPC, MoA, MoPS and SFDA	2003	Updates of rabies situation in China
Rabies Prevention and Treatment Standard	MoA	2002, revised in 2006	Technical guideline for animal rabies control and prevention (diagnoses, case definition, reporting and animal disposal)
Animal Diseases Surveillance System	MoA	2009	Requirements for animal disease surveillance, for which rabies is only a part of it
National medium and long-term animal epidemic prevention planning (2012–2020)	State Council	2012	Objectives (indicators) for rabies control by 2015 and 2020 - No indications on strategies to reach these objectives

*Ministry of Agriculture (MoA); National Health and Family Planning Commission (NHFPC); Ministry of Public Security (MoPS); State’s Food and Drugs Administration (SFDA).

All review articles [15,16,19,28-31] showed a dearth of knowledge about rabies in the general public and among health workers – particularly in rural areas. This was based on patchy data collected in limited areas. Si H *et al.* reported rabies-related information provided to the public is mainly concentrated in municipal or district Center for Disease Control (CDC)s as opposed to less dissemination in other public settings such as community hospitals, clinics, townships, villages, and police stations [31]. In rural Guizhou or Guangdong provinces poor understanding of rabies among the public persists: only 15% would clean and disinfect wounds, and 23% would seek medical treatment rabies clinics following a dog bite. In contrast, high post-exposure vaccination coverage among patients bitten by dogs is present in some of the major cities of the coastal areas (e.g. in Shanghai, 99%).

### Control in humans

In recent years a series of technical protocols on surveillance, treatment and diagnostic of rabies has been introduced or updated [32-34]. Despite progress in standardizing PEP across the country, authors underscored some implementation gaps: although the standard post-exposure vaccination schedule i.e. Zagreb regimen of rabies vaccine has been approved by the State Food and Drug Administration of China and used in China since 2010, other schedules may still be used [31]. In China, inadequate PEP (incomplete and improper PEP) and some evidence of low quality vaccines have been documented [31]. Seroconversion testing in patients is still performed in many places although it is not recommended for patients completing pre-exposure or PEP [16,35]. For instance the national policy [32] required for instance reasonable availability of PEP clinics – notably in remote areas; unfortunately information is unavailable regarding effective implementation of this requirement. One way to reduce financial cost for many who cannot afford PEP is to adopt an intra-dermal regimen, which is treatment recommended by the World Health Organization (WHO) that has been demonstrated to be as effective as that of intra-muscular administration [36].

According to the 2009 Ministry of Health statistics, China administers 12–15 million rabies vaccine doses annually at a cost of about US$ 1 billion, making it the world’s largest market. A PEP (~US$ 185/person) and Rabies Immunoglobulin (RIG) treatment costs are beyond the reach of most rural residents [22]. Besides Sanofi and Novartis which registered human rabies vaccines in 1999, there are presently several locally registered manufacturers (n = 13). RIG was first registered in 1994 and there are currently 15 registered manufacturers. With the rapid income growth of the Chinese middle class and the fear of rabies, demands for efficient vaccines and their related societal costs will continue to rise unless rabies is controlled in domestic dogs, a situation that has been seen elsewhere in the region (e.g. Vietnam, Thailand, Sri Lanka) [37].

### Control in animals

The data on dog vaccination rates from a survey in 15 sentinel counties estimated the annual average dog vaccination coverage varied from 1.6% to 20.4% for a dog to human ratios of 10.2:100 – 21.8:100 between 2006 and 2009 [38]. However, large disparities exist for these estimates between rural areas and cities or western and coastal provinces. Recent canine vaccination coverage is as high as 93% in Shanghai and 77% in some cities of Shandong province (a wealthy coastal province) [21] as opposed to 4% - 10% in endemic rural areas of Guizou province [30,31].

Despites the existence of national guideline on rabies control in dogs [39], the country has only enforced national immunization in dogs since 2008 [40]. However, the owners bear the responsibility of the payment of registration and vaccination [41]. Dogs are registered at local police departments, while the rabies vaccines are administrated by Veterinary Services. A coordination mechanism has been proposed but there is no real evidence it is working. Doubts persist regarding the quality of vaccines manufactured in China while officials were concerned about shortages of a canine rabies vaccine required to meet China’s need for adequate coverage of its dog population. To date, 20–40 million doses of attenuated vaccines are produced annually, which mainly meet the demands of large cities [41].

### Surveillance in humans and dogs

Human rabies is a notifiable disease in China. Since 2003, China’s surveillance for infectious diseases has improved greatly. Surveillance is systematic and nationwide using an innovative hospital and web-based reporting system [42]. In 2005, 15 sentinel counties of six endemic provinces were identified for enhanced rabies surveillance activities which included case reports, post-exposure prophylaxis and laboratory testing in humans, and animal reservoir and dog vaccination rates [43]. However, under-reporting and under-recognition occur, which is an inherent problem with any passive surveillance. China CDC also recognizes that many people living in poverty or in rural areas has difficult access to public health services, and that cases might not be properly reported as many peripheral health facilities have no internet access. Underreporting may be higher when it comes to rabies as most victims live in remote and poor areas; it was found to be >35% based on a capture-recapture study conducted in 2006 in Hunan province [44]. Human rabies is mainly clinically diagnosed. Of 10,866 reported rabid cases during 2005–2010 only 238 (2.2%) were laboratory-confirmed.

MoA adopted a national protocol for national rabies surveillance in dogs in 2009 [45]; however, for 1,911 human cases detected in China in 2011, only seven rabid dogs were reported, indicating that diagnosis and surveillance for animal rabies is not functioning optimally across the country (Tu C, unpublished observation). Joint investigations between public health, veterinary and public security services are rarely seen (FAO, unpublished observation).

## Discussion

Our review was unique in attempting to highlight the strengths and gaps in the rabies control program in China so that potential control activity and related complications could be addressed. Considerable progress has been made to curb the re-emergence of rabies in China since 2005. As a result, the incidence has declined steadily since the peak in 2007. Reasons for this decrease are likely explained by the country’s investment in training health professionals on PEP and increasing access to PEP in the countryside (Yin W’s unpublished observation). There are few reports of significant investments from local governments of wealthy cities in endemic areas (e.g. Dongguan, Guangdong province) for free dog vaccination campaigns and rural domestic dogs (Tu C, unpublished observation). Other provincial governments (e.g. Guizhou, Anhui) have recently adopted a health insurance system for rural populations to partially cover PEP costs, although reimbursement rates (~US$ 15 per complete PEP) is recognized to be largely insufficient [46].

Chinese authorities at the highest level have identified rabies as a priority to be targeted with a series of progressive objectives until 2020 [25]. More significantly, the public health authorities have recognized that preventing human rabies requires rabies control in the dog population. As such, China has passed policies supporting intersectoral collaborations between ministries and departments involved in the management of the dog population [15]. Technical departments of NHFPC and MoA have paid particular attention to establishing national norms and standards for rabies control and prevention. In addition, they have developed technical guidance by strengthening the laboratory capacity for rabies diagnostics to all provincial public health laboratories in endemic zones. A contributing factor to the potential success of a control program is the existence of a national supply of vaccines for humans and animals as well as human/equine immunoglobulins. It is also crucial for the government to negotiate with local vaccine manufacturers for affordable prices and guaranteed orders of significant amounts of vaccine [47,48].

Despite the increasing volume of publications, legislation and regulations in the recent years, our review points to some major information gaps that provide insight into the level of effectiveness of a nationwide rabies control program in a long run. Undeniably, Chinese policymakers’ commitment to control rabies has been a substantial achievement; however, there is a concern that this commitment will fade rapidly when reports of human rabies decrease to a certain level, a situation that already occurred in the late 1980’s [15]. We believe that additional evidence is needed to sustain a serious commitment. Indeed, there is a paucity of data regarding the burden of rabies in China, which include estimates of the true incidence and on the relative cost of PEP or disease for the families (e.g. costs of travel for several visits and medical fees) and society. Rabies is thought to be commonly under-reported or under-recognized, resulting in the under-estimate of the true disease burden. Notably, one published households survey on child injury conducted among 319,543 people including 98,335 children aged <18 years in Jiangxi province in 2007 estimated that 89 children may have died of animal (mainly dogs) bites while China CDC reported 21 rabies cases (i.e. fourfold underreporting) from the same year and same age group [2,49]. Disease Burden data would help provide robust estimates of the cost-benefit ratio of a canine elimination program and accurately determine the most cost-effective strategies for China using a large variety of available options (e.g. free PEP, free dog vaccination, dog population management strategies, decentralization of control, …etc.) [50].

The second major finding is the presence of information gaps regarding health services or health systems research, economic evaluations, and research that focuses on evidence-based control practices. When it comes to research to improve existing interventions, publications were skewed towards laboratory and virology related works (i.e. new vaccines or diagnostics development). Although connecting researchers with policy makers and control program implementers always has been challenging [51], our review showed that the knowledge generated is non-existent, inferior, or of variable quality, particularly that which could lead to practical action for rabies control. We are under the impression that provincial and more peripheral Bureaus of Health are left to organize improvised control strategies including some that had little evidence for efficiency. Operational research that brings about evidence from the field and could influence practical actions to improve prevention, control and elimination should be encouraged. Examples illustrating the lack of operation-related data include (1) precise estimates and mapping of dog population size/density and their profiles (e.g. rates of roaming or stray dogs) at the most peripheral level, a condition which demonstrates impact (vaccination coverage), develops an effective design and planning of a dog vaccination campaign, and motivates policymakers; and (2) a reduction of human case reports has been observed, however, the challenge is with the NHFPC’s ability to measure the impact of interventions that were introduced. Data are lacking to monitor access and quality of PEP on a routine basis. In other words, monitoring and evaluation mechanisms are needed as they become the essential tools for detecting, measuring and interpreting changes overtime. They identify the impact of management activities and subsequent alteration for improvement; (3) developing a research agenda to appropriately inform the policymakers as well as the program managers (e.g. cost-effective strategies for dog vaccination and birth control; education and health promotion on rabies targeting children, development of tools and instruments; and an effective strategy for dog population management adapted to local resources and specificities); (4) addressing the issue of a potential sustained rabies wildlife rabies cycle. This highlights the importance of considering some locations for specific surveillance. Only when rabies in dogs is controlled, we can better understand the scale of rabies in wildlife.

Overall, there is a need for more strategic planning to provide a sense of direction and involve concerned people and groups. More importantly this is preparation for progressive steps from uniting the conditions for success (securing funds, correcting the provision of licensed vaccines for humans and dogs, assuring structural changes, raising awareness and community participation, etc.), and designing a roadmap to scaled- up interventions towards a common goal (i.e. elimination of human rabies in China). We also highlight the need for a research agenda addressing the operational needs and constraints in China. In short, this strategy should be a multisectoral effort which is reflected in the form a national field manual for rabies elimination for both the public health and animal health sectors. Such a document should be an evolving guideline that could be initiated based on already existing guiding principles [52] and lessons learned from the field or other countries which are currently engaging in rabies elimination. Furthermore, this document could serve as a model for a long-lasting WHO and FAO’s agenda for the “One-Health” concept, a concept that advocates for a close collaboration between the medical and veterinary professions in industrialized and lower-income countries [53,54].

## Conclusion

China has reduced substantially rabies incidence since 2007; however, the authorities’ commitment may fade rapidly when reports of human rabies decrease to a certain level. We believe that additional evidence is needed to sustain commitment. The published articles review showed the contrast between an emphasis on knowledge-driven research and the absence of information for effective policies. Monitoring and evaluation mechanisms are lacking to appropriately inform and improve disease control programs. We advocate for strategic planning to address program gaps within the framework of a rabies elimination goal.

## Competing interests

The authors declare that they have no competing interests.

## Authors’ contributions

WY and SV carried out the study design, writing and proofreading. JD and HZ carried out the data collection and analysis and draft of the manuscript. CT, JE, FG and HY participated in the provision of data, discussion and review. All authors read and approved the final manuscript.

## Supplementary Material

Additional file 1 Multilingual abstracts in the six official working languages of the United Nations. Click here for file
