# Supplementary material for: Challenges and needs for China to eliminate rabies
Source: Infect Dis Poverty. 2013 Oct 2;2:23. doi: 10.1186/2049-9957-2-23 (PMC3851465; doi:10.1186/2049-9957-2-23)
Supplement: Additional file 1 — Multilingual abstracts in the six official working languages of the United Nations. [file 2049-9957-2-23-S1.pdf]

Translation of the abstract into the six official working languages of the United Nations

التحديات والاحتياجات للقضاء على داء الكلب في الصين

ون وو يين، جي دونج، تشانجتشون تو، جون إدواردز، فوشينج جوا، هانج زيو، هونججيه يو، سيرندا فونج

الملخص

في الصين، يُعتبر داء الكلب مصدراً كبيراً للقلق بالنسبة الصحة العامة حيث تبقى الكلاب هي الحاضن الرئيسي لانتقال المرض إلى البشر؛ كما تُعد الوفيات المرتبطة بداء الكلب في المرتبة الثانية في العالم.

لقد قُمنا بتجميع كل المقالات التي نُشرت والوثائق الرسمية على داء الكلب في البر الرئيسى للصين لدراسة التحديات والاحتياجات من أجل القضاء على داء الكلب في البلاد. وقد حددت السلطات الصينية داء الكلب بأنه ذو أولوية، كما صرحت بأن مكافحة داء الكلب في الكلاب هو مفتاح السيطرة على داء الكلب في البشر وأوضحت أن ضرورة وأهمية التعاون بين القطاعات المختلفة. وقد بذلت جهود للاستجابة بفعالية لعودة ظهور داء الكلب، والتي بلغت ذروتها في عام 2007 بتشخيص أقل من 3،300 حالة. وعلى الرغم من هذه النتائج والأحجام المتزايدة من النشرات واللوائح في السنوات الأخيرة، إلا أن دراستنا أشارت إلى بعض الثغرات في البيانات الرئيسية لتحسين أنشطة مكافحة داء الكلب ووضع تصور لبرنامج التخلص من هذا الوباء. وقد يتعارض التركيز في الدراسات السابقة سواء في المختبرات أو في الأبحاث الوبائية المرتبطة أو حتى أبحاث الأوبئة الأساسية مع غياب المعلومات أو البيانات اللازمة لرصد الأنظمة المُختلفة في الجنس البشري وفي الحيوانات (مثل جودة المراقبة والاستجابة والوقاية بعد التعرض للمرض). كما يُوجد أيضاً نقص في وسائل إحاطة صناع القرار بشكل مناسب (مثل العبء الاقتصادي للمرض، وتأثير السياسات)، ومساعدة مديري البرامج (على سبيل المثال التوجيه الشامل والإرشاد الاستراتيجي لأنشطة الوقاية الفعالة من حيث التكلفة والسيطرة، والتعليم العام والتوعية وإدارة أعداد الكلاب).

في الختام، لا بد من التخطيط الاستراتيجي لتوفير التوجه العلمي إلى الهدف، وتوضيح أهمية إثبات جدوى القضاء على داء الكلب في الصين، ووضع جدول أعمال للأبحاث، يتناول كيفية معالجة الاحتياجات والقيود التشغيلية في البلاد. كما يجب أن يكون التخطيط هو ناتج جُهد القطاعات المُختلفة.

Translated from English version into Arabic by Freelanceabunar, through

TRANSLATORS
WITHOUT BORDERS

中国消除狂犬病的挑战和需求

殷文武，董婕，涂长春，John Edwards，郭福生，周航，余宏杰，Sirenda Vong

摘要：

在中国, 狂犬病是一个公共卫生重点关注的传染病，其传播宿主动物主要是狗，狂犬病年死亡人数位居世界第二。我们检索整理了中国大陆发表的关于狂犬病文献和官方文件, 试图找到中国狂犬病消除所面临的挑战和需求。中国政府高度重视狂犬病的防控，已经认识到狂犬病的控制有赖于多部门合作，狗的管理是控制人间狂犬病的重要措施。面对近年来狂犬病的再流行（2007 年达到高峰，一年的病例数超过 3300 例），中国政府努力采取一系列措施努力应对，已经取得了显著的效果。尽管近年来取得一些防控效果，越来越多的文献发表和政策法规的出台，我们的综述发现对于改进狂犬病控制乃至消除项目仍然存在信息上的不足。在文献中我们可以看到大部分研究集中在实验室技术、病原学研究以及基础流行病学，与此形成鲜明对比的是缺乏对人和动物系统的动态评估(如疫情的监测与应对、暴露后处置的质量)，缺乏对决策者适宜的信息（如疾病的经济负担、对政治的影响），也缺乏对项目管理者适宜的提示（如考虑成本效益的防控活动，大众教育，狗的数量控制）。总之，需要加强部门间合作, 制定战略规划, 为中国的狂犬病消除提供目标和可行性论证，制定研究日程，强调实际的需求和挑战。

Translated from English version into Chinese by Dong Jie

Élimination de la rage en Chine : défis et besoins

Wenwu Yin, Jie Dong, Changchun Tu, John Edwards, Fusheng Guo, Hang Zhou, Hongjie Yu, Sirenda Vong

Extrait

En Chine, la rage constitue un grave problème de santé publique, dans le cadre duquel les chiens demeurent le principal réservoir de transmission de la maladie aux humains ; le pays arrive au deuxième rang mondial de la mortalité liée à la rage.

Nous avons regroupé tous les articles et documents officiels publiés au sujet de la rage en Chine continentale afin d'examiner les défis et les besoins en matière d'élimination de la maladie dans le pays. Les autorités chinoises ont établi que la rage était une priorité, ont reconnu le contrôle de la rage chez les chiens comme essentiel pour contrôler la rage chez les humains et ont exigé des collaborations intersectorielles. Des efforts ont été faits pour répondre efficacement à la dernière réapparition de rage, qui a atteint un pic de plus de 3 300 cas en 2007. Malgré ces résultats et le volume de publications et de règlements en hausse au cours des dernières années, notre examen met en lumière certaines lacunes majeures sur le plan de l'information en vue d'améliorer les activités de contrôle de la rage et d'envisager un programme d'élimination. L'accent mis sur la recherche épidémiologique en laboratoire, ou associée aux pathogènes et de base dans la littérature contraste avec l'absence d'information permettant de surveiller divers systèmes chez les humains et chez les animaux (p. ex., qualité de la surveillance, intervention et prophylaxie post-exposition). Les renseignements sont également insuffisants pour informer les décideurs (p. ex., fardeau d'une maladie sur l'économie, répercussions des politiques) et aider les gestionnaires de programmes (p. ex., orientation stratégique exhaustive aux fins de prévention et d'activités de contrôle rentables, éducation du public et gestion de la population canine) de façon appropriée.

Enfin, une planification stratégique est nécessaire pour donner un sens de l'orientation, prouver la faisabilité de l'élimination de la rage de Chine, et mettre sur pied un programme de recherche, qui réponde aux besoins et aux contraintes opérationnels du pays. Cette planification devrait être le fruit d'un effort multisectoriel.

Translated from English version into French by Mélanie Bernard, through

TRANSLATORS
WITHOUT BORDERS

Проблемы и потребности борьбы с бешенством в Китае

Вэньу Инь, Цзе Дун, Чанчунь Ту, Джон Эдвардс, Фушэн Го, Хан Чжоу, Хунцзе Юй, Сиренда Вон

Аннотация

Одной из важнейших проблем здравоохранения в Китае является бешенство, передающееся человеку главным образом через собак. По уровню смертности от бешенства Китай занимает второе место среди стран мира.

Для настоящей работы мы собрали и изучили все публикации и официальные документы на тему бешенства на основной территории Китая с целью выявить потребности в борьбе с бешенством в стране и сопутствующие этому трудности. Китайские власти признают, что борьба с бешенством является одной из приоритетных задач государства и что ключевой задачей в этой связи является контроль состояния собак. При этом власти настаивают на необходимости установления межотраслевого сотрудничества. Потребность в принятии действенных мер была вызвана недавним всплеском данного заболевания в стране, пик которого пришелся на 2007 год – более 3 300 случаев. Несмотря на полученные результаты и на возросший объем публикаций и законодательных актов в данной области в последние годы наше исследование выявило ряд важных информационных упущений, которые необходимо восполнить с целью улучшения мер по контролю заболеваемости и составления программы по уничтожению бешенства. В литературе достаточно подробно описаны лабораторные или патогенные и основные эпидемиологические исследования, однако при этом отсутствуют данные по мониторингу различных человеческих и животных систем (такие как качество надзора, ответных мер и последующей профилактики). Также существует нехватка сведений для надлежащего информирования директивных органов (данные об экономическом бремени, эффективности принимаемых мер и т.д.) и содействия руководителям программ (комплексные и стратегические рекомендации по экономичным мерам профилактики и контроля, просвещению общественности и контролю популяции собак и т.д.).

Таким образом, необходимо стратегическое планирование, которое поможет определить направление, докажет осуществимость мер по искоренению бешенства в Китае и позволит составить план исследования для определения существующих в стране оперативных нужд и ограничений. Для этого требуются совместные усилия специалистов в различных отраслях.

Translated from English version into Russian by Chrisfly, through

TRANSLATORS
WITHOUT BORDERS

Desafíos y necesidades para eliminar la rabia en China

Wenwu Yin, Jie Dong, Changchun Tu, John Edwards, Fusheng Guo, Hang Zhou, Hongjie Yu, Sirenda Vong

Resumen

En China, la rabia es un asunto de sanidad pública en el que los perros siguen siendo la fuente principal de transmisión de enfermedades a los humanos; la tasa de mortalidad relacionada con la rabia ocupa el segundo lugar en el mundo.

Hemos recopilado todos los artículos publicados y documentos oficiales sobre la rabia en China para examinar los desafíos y necesidades para eliminar la rabia en el país.Las autoridades chinas han identificado a la rabia como una prioridad, y ha reconocido que el control de la rabia en los perros es clave para controlar la rabia en los seres humanos, lo que requiere la colaboración de distintos sectores.Se han realizado esfuerzos para responder con eficacia al último brote de rabia, que alcanzó su punto álgido en 2007 con más de 3,300 casos.Pese a estos resultados y el creciente volumen de publicaciones y regulaciones de los últimos años, nuestro análisis señala algunas brechas de información importantes para mejorar las actividades de control de la rabia y concebir un programa de eliminación.Un énfasis en las investigaciones de laboratorio, o asociadas con patógenos, e investigaciones básicas de epidemiología en los textos, ha mostrado un contraste con la falta de información para monitorizar distintos sistemas en humanos y animales (p. ej. la calidad de la vigilancia, la respuesta y la profilaxis post-exposición).También falta información para poner al corriente a los legisladores de manera adecuada (p. ej. la carga económica de la enfermedad, el impacto en las políticas) y asistir a los gestores de programas (p. ej. con una dirección exhaustiva y estratégica para las actividades de prevención rentables y de control, educación pública y gestión de la población canina).

En resumen, se necesita planificación estratégica para ofrecer un sentido de dirección, demostrar viabilidad de la eliminación en China, y desarrollar un plan de investigación que aborde las necesidades operativas y limitaciones del país .La planificación debe ser un esfuerzo realizado por múltiples sectores.

Translated from English version into Spanish by Adriana Acevedo, through

TRANSLATORS
WITHOUT BORDERS
